# Prevalence and Associated Factors of Schistosomiasis among Children in Yemen: Implications for an Effective Control Programme

**DOI:** 10.1371/journal.pntd.0002377

**Published:** 2013-08-22

**Authors:** Hany Sady, Hesham M. Al-Mekhlafi, Mohammed A. K. Mahdy, Yvonne A. L. Lim, Rohela Mahmud, Johari Surin

**Affiliations:** 1 Department of Parasitology, Faculty of Medicine, University of Malaya, Kuala Lumpur, Malaysia; 2 Department of Parasitology, Faculty of Medicine and Health Sciences, Sana'a University, Sana'a, Yemen; 3 Research Department, University of Science and Technology, Sana'a, Yemen; Jiangsu Institute of Parasitic Diseases, China

## Abstract

**Background:**

Schistosomiasis, one of the most prevalent neglected tropical diseases, is a life-threatening public health problem in Yemen especially in rural communities. This cross-sectional study aims to determine the prevalence and associated risk factors of schistosomiasis among children in rural Yemen.

**Methods/Findings:**

Urine and faecal samples were collected from 400 children. Urine samples were examined using filtration technique for the presence of *Schistosoma haematobium* eggs while faecal samples were examined using formalin-ether concentration and Kato Katz techniques for the presence of *S. mansoni*. Demographic, socioeconomic and environmental information were collected via a validated questionnaire. Overall, 31.8% of the participants were found to be positive for schistosomiasis; 23.8% were infected with *S. haematobium* and 9.3% were infected with *S. mansoni*. Moreover, 39.5% of the participants were anaemic whereas 9.5% had hepatosplenomegaly. The prevalence of schistosomiasis was significantly higher among children aged >10 years compared to those aged ≤10 years (*P*<0.05). Multivariate analysis confirmed that presence of other infected family member (*P*<0.001), low household monthly income (*P* = 0.003), using unsafe sources for drinking water (*P* = 0.003), living nearby stream/spring (*P* = 0.006) and living nearby pool/pond (*P* = 0.002) were the key factors significantly associated with schistosomiasis among these children.

**Conclusions/Significance:**

This study reveals that schistosomiasis is still highly prevalent in Yemen. These findings support an urgent need to start an integrated, targeted and effective schistosomiasis control programme with a mission to move towards the elimination phase. Besides periodic drug distribution, health education and community mobilisation, provision of clean and safe drinking water, introduction of proper sanitation are imperative among these communities in order to curtail the transmission and morbidity caused by schistosomiasis. Screening and treating other infected family members should also be adopted by the public health authorities in combating this infection in these communities.

## Author Summary

Schistosomiasis remains one of the most serious and prevalent diseases worldwide. Despite intensive control efforts by the government and international bodies, schistosomiasis is the second cause of death, after malaria, in Yemen, with an estimated 3 million cases. We screened 400 children in rural areas of five provinces in Yemen for the presence of schistosomiasis. Overall, 31.8% of the children were found to be positive for schistosomiasis; 23.8% were infected by *Schistosoma haematobium*, and 9.3% were infected by *S. mansoni*. The study identified the presence of other family members infected with schistosomiasis, low household monthly income, using unsafe water supply as a source for drinking water, living nearby stream/spring and/or pool/pond as the key factors significantly associated with schistosomiasis in these communities. Innovative and integrated control measures to control this infection should be implemented among this population. Periodic school-based and community-based drug distribution, health education, provision of clean and safe drinking water, introduction of proper sanitation will help to reduce the prevalence and morbidity of schistosomiasis among these communities.

## Introduction

Schistosomiasis or bilharzia, one of the most prevalent neglected tropical diseases (NTDs), is still a public health problem in many developing countries in the tropics and subtropics with approximately 240 million infected people and about 700 million people worldwide are at risk of this infection [1]. Over 90% of the disease is currently found in sub-Saharan Africa, where more than 200,000 deaths are annually attributed to schistosomiasis, and Middle East and North Africa regions [2]–[4]. Despite intensive efforts to control the disease, schistosomiasis together with soil-transmitted helminthiasis continue to represent more than 40% of the disease burden caused by all tropical diseases, excluding malaria [5].

Schistosomiasis is mainly caused by three different species of blood-dwelling fluke worms of the genus *Schistosoma* namely *Schistosoma haematobium* (causes urinary schistosomiasis), *S. mansoni* and *S. japonicum* (both cause intestinal schistosomiasis). Clinical manifestations of schistosomiasis are associated with the species-specific oviposition sites and the burden of infection [6]. Urinary schistosomiasis is characterized by haematuria as a classical sign. It is associated with bladder and uretral fibrosis, sandy patches in the bladder mucosa and hydronephrosis that are commonly seen in chronic cases while bladder cancer is possible as late stage complication [7]. On the other hand, intestinal clinical manifestations include abdominal pain, diarrhea, and blood in the stool. In advanced cases, hepatosplenomegaly is common and is repeatedly associated with ascites and other signs of portal hypertension [8], [9].

Among the Middle East countries, Yemen has the highest percentage of people living in poverty where more than 50% of the population of nearly 25 million people lives below the poverty line [10]. The country has been unstable for several years, suffering from civil wars, a deteriorating economy and severe depletion in water resources. With regards to NTDs, Yemen is endemic for at least 8 NTDs namely soil-transmitted helminthiasis, schistosomiasis, onchocerciasis, lymphatic filariasis, leishmaniasis, fascioliasis, trachoma and leprosy. Moreover, the country ranks first in trachoma; second in schistosomiasis, ascariasis, fascioliasis and leprosy; and fourth in trichuriasis and cutaneous leishmaniasis [4].

In 2008, Yemen launched its first campaign to eliminate schistosomiasis as a national public health problem with the aim of eliminating schistosomiasis-related morbidity through annual treatment to school-age children with a financial support from the World Bank and World Health Organization (WHO) [11]. Despite of these support and efforts to control the disease in Yemen, the prevalence of schistosomiasis remains largely unchanged (since 1970s) with prominent morbidity [12]–[17]. Moreover, new foci of schistosomiasis transmission have been identified.

Hence, the aims of the present study were to determine the prevalence and distribution of schistosomiasis and to identify the associated key factors of this disease among Yemeni children in rural areas which are undergoing active control and prevention surveillances. It is hoped that findings of this study will assist public health authorities to identify and implement integrated and effective control measures to reduce the prevalence and burden of schistosomiasis significantly in rural Yemen.

## Materials and Methods

### Ethical statement

The study protocol was approved by the Medical Ethics Committee of the University of Malaya Medical Centre (Ref. no: 968.4). It was also approved by the Hodeidah University, Yemen and permission to start data collection was also given by the Yemen Schistosomiasis National Control Project. The head of households and children were informed about the study objectives and methods and the priority of the consent for inclusion of children. Moreover, they were informed that they could withdraw their children from the study without any consequences. Thus, written and signed or thumb-printed informed consents were obtained from all adult participants before starting the survey. Similarly, written and signed or thumb-printed informed consents were taken from parents or guardians, on behalf of their children. All the infected children were treated with a single dose of 40 mg/kg body weight praziquantel tablets. Each child swallows the tablets with some water, while being observed by the researcher and medical officer (Direct Observed Therapy) [18].

### Study design

A cross-sectional community-based study was carried out among children aged ≤15 years in rural areas in Yemen. Data were collected in a period of seven months from January to July 2012. In each province, two rural districts were selected randomly from the available district list and then two villages within the selected districts were considered in collaboration with the Schistosomiasis Control Project office in each province. The number of inhabitants per household was recorded and all of them were invited to participate in this study. Unique reference codes were assigned to each households and study participants.

### Study area

This study was carried out in five provinces in Yemen namely Taiz, Ibb, Dhamar, Sana'a and Hodiedah. These provinces are endemic for schistosomiasis and undergoing active surveillances by the schistosomiasis national control project. The highest prevalence of schistosomiasis was reported in Hajjah and Taiz provinces [15], [17]. However, we could not collect samples from Hajjah during the sampling period due to civil war which occurred in 3 provinces including Hajjah.

Sana'a and Dhamar represent the mountainous areas at an altitude of >2000 m above sea level with a total population of 4 million. Taiz, Hodiedah and Ibb represent the country's coastal plains and foothills at an altitude of <2000 m above sea level with a total population of 6.5 million. In Yemen, climate varies from hot and high humidity in the coastal areas to cold in the highlands. In the coastal areas, relative humidity ranges between 70% and 90% and mean annual rainfall is about 200 mm with two rainy seasons (February–April and July–September). In the highlands, the relative humidity ranges between 20% and 50%, mean annual rainfall is about 800 mm, and the climate is moderate in summer and cold in winter.

**Figure 1:**
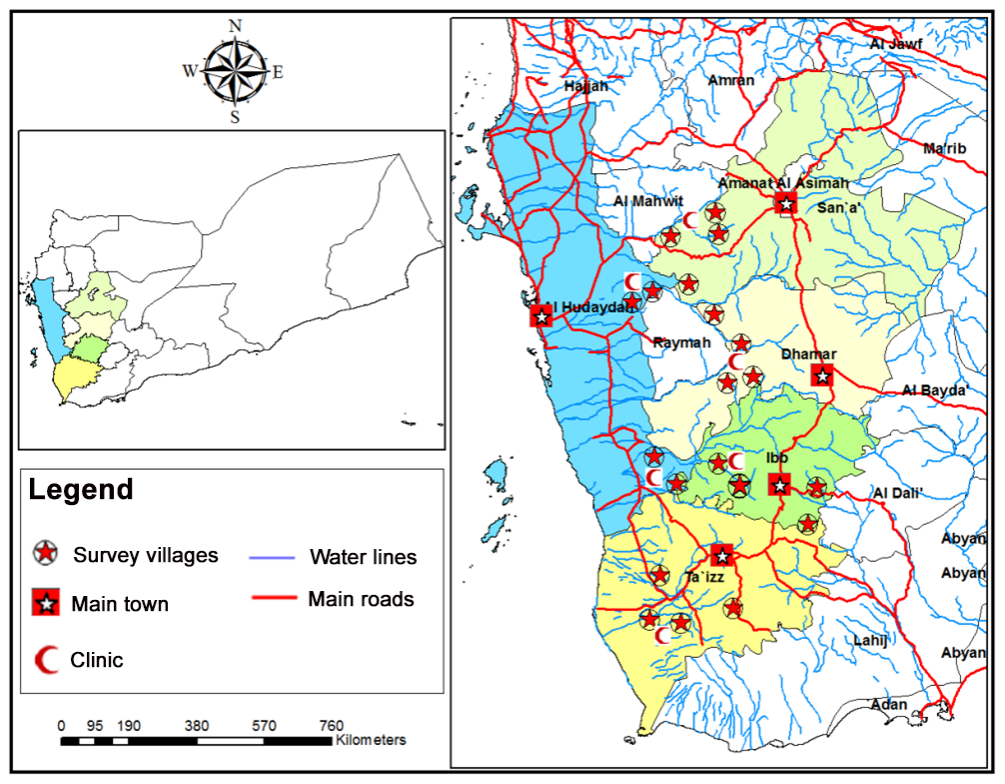
A geographic map showing study area in 5 provinces in Yemen.

Ten districts were selected for this study namely Mosa and Almafer (Taiz), Alsabrah and Alodien (Ibb), Otmah and Gabal al sharq (Dhamar), Alhemah and Manakhah (Sana'a), and Gabal Ras and Bora (Hodiedah) (Figure 1). The inclusion criteria in selecting these study areas were rural areas and undergoing active control surveillance. Moreover, the selection process was done after discussion with the schistosomiasis national control project personnel. Rural areas are farmlands which depend on streams, underground wells and rain (water tanks) as the main source of water for domestic and irrigation purposes. Agriculture is the main occupation of the people in these areas and surface traditional irrigation system is still dominant and covers large agricultural areas creating favorable snail-breeding conditions. Snail populations of different genera were identified in different water sources at the study areas and heavily infested water sources were observed.

### Study population

**Figure 2:**
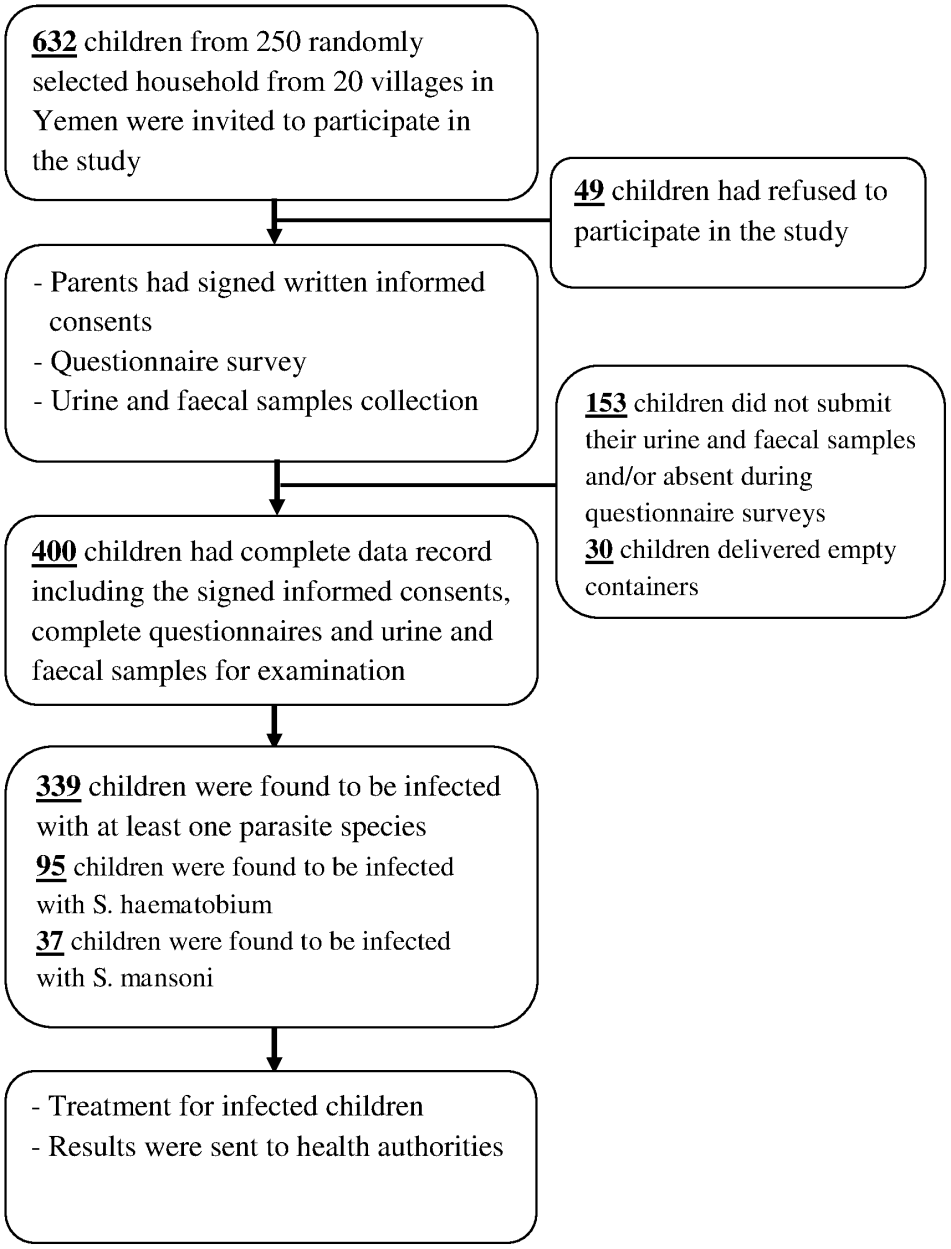
Flow chart of the participation and compliance in the present study.

Out of about 780 households, 250 households were selected randomly from the villages for this study. We attempted to enrol all available children ≤15 years of age from the selected households after acquiring consent from the head of the households. Although 632 children received stool and urine containers, only 430 (68.0%) children delivered the containers for examinations. In this study, 202 (32.0%) failed to submit samples and/or absent during questionnaire surveys and 30 (4.7%) containers were returned empty. Hence, they were excluded from the study. Overall, 400 (63.3%) children (59.5% males and 40.5% females) who had delivered suitable samples for examination with complete questionnaire data were included in this study (Figure 2). Throughout many visits to the study areas, most of the children were observed to play outside without wearing shoes or slippers. Some of the children play and swim in the streams/pools after school and in their leisure time. Besides that, their personal hygienic practices were also poor. Poverty prevails at these areas with very poor housing and living conditions and was notably observed in areas from Hodeidah province.

### Questionnaire survey

A pretested questionnaire was used to collect data about the demographic, socio-economic, environmental background, personal hygiene, clinical signs and symptoms of urinary or intestinal schistosomiasis and history of receiving anti-schistosomal treatment. Information was collected from the children's parents or adult guardians via face-to-face interview. In addition, participants underwent physical examination and direct observation in order to record more details about their weight, height, body temperature, hepatosplenomegaly and personal hygiene. During the interviews, direct observation was made by an assistant on the personal hygiene of the children and household cleanliness including the availability of functioning toilets, piped water, cutting nails, wearing shoes when outside the house and washing hands.

### Parasitology

Faecal and urine samples were collected from each subject, between 10 am and 2 pm when maximum eggs excretion occurs [19], into 100 mL clean containers with wide mouth and screw-cap. The containers were placed into zipped plastic bags, kept in a protected ice box and transported for examination at the nearest health center laboratory within 4 hours of collection.

The faecal samples were examined using Kato-Katz technique for the presence of *S. mansoni* eggs [18]. Negative faecal samples were re-examined by formalin ether sedimentation technique as described by Cheesbrough [20] before the negative results were confirmed. To determine the worm burden, egg counts were taken and recorded as eggs per gram of feces (epg) for each positive sample and the intensity of infections was graded as heavy, moderate or light according to the criteria proposed by the WHO [18]. On the other hand, urine samples were examined for the presence of *S. haematobium* eggs by filtration method using nucleopore membrane. Besides that, dipstick test was also used [21]. For quality control, urine and faecal samples examination were performed in duplicate for about 25% of the samples, selected randomly.

### Haemoglobin measurement

A finger prick blood was obtained from each child and hemoglobin (Hb) level was assessed directly by using the HemoCue hemoglobinometer (HemoCue, AB, Angelhom, Sweden). Children with Hb levels lower than 12 g/dl were considered to be anaemic [22].

### Statistical analysis

Data was double-entered by two different researchers into Microsoft Office Excel 2007 spreadsheets. Then, research leader cross-checked the two data sets for accuracy and created a single data set. Data analysis was performed by using *Statistical Package for Social Sciences for Windows* (SPSS) version 18. Presence of schistosomiasis, demographic, socioeconomic, environmental and behavioural characteristics were treated as categorical variables and presented as frequencies and percentages. For inferential statistics, the dependent variable was schistosomiasis whereas the independent variables were the demographic factors (age and gender), socioeconomic factors (fathers' educational levels, parents' employment status, household monthly income, family size) and environmental factors (sources of drinking water, sources of household water, presence of streams, dams, wells, ponds, tanks, pools, troughs or any other man-made water collection places). Chi-square test was used to examine the significance of the associations and differences in frequency distribution of variables. Odd ratios (OR) and 95% confidence intervals (CI) were computed.

Multiple logistic regression analysis was used to identify the factors significantly associated with schistosomiasis; OR and its corresponding 95% CI were calculated based on the final model. All variables that showed significant difference with *P*≤0.25 in the univariate analyses were used to develop the multiple logistic regression “STEPWISE” models as suggested by Bendel and Afifi [23]. All tests were considered significant at *P*<0.05.

## Results

### General characteristics of study population

Four hundred children aged ≤15 years with a mean age of 10 years (95% CI = 9.7, 10.2) participated voluntarily in this study. The general characteristics of the participants and their families are shown in Table 1.

**Table 1:** General characteristics of Yemeni children who participated in this study (n = 400).

Characteristics	n (%)
**Age groups (years)**	
≤10	222 (55.5)
>10	178 (44.5)
**Gender**	
Males	238 (59.5)
Females	162 (40.5)
**Residency**	
Sana'a	77 (19.3)
Taiz	76 (19.0)
Ibb	69 (17.3)
Hodiedah	85 (21.3)
Dhamar	93 (23.3)
**Socioeconomic status**	
Fathers' education level	
Not educated	191 (47.8)
Primary school	104 (26.0)
Secondary school	78 (19.5)
University	27 (6.8)
Fathers' occupational status	
Government employees/professionals	171 (42.7)
Farmers	195 (48.8)
Not working	34 (8.5)
Working mothers	21 (5.3)
Low household income (<YER 20,000)	237 (59.3)
Large family size (≥8 members)	210 (52.5)
Piped water supply	87 (21.8)
Electricity	111 (27.8)
Presence of toilet in house	168 (42.0)
**Health status**	
Anaemia	158 (39.5)
Hepatosplenomegaly	37 (9.3)
Intestinal Parasitic infections (at least one species)	339 (84.8)

All values are number (%). YER, Yemen Rial; (US$1 = YER 214).

Overall, almost half of the fathers had no formal education and about half (48.8%) of them were farmers whereas 42.7% were government employee and/or professionals. On the other hand, almost all the mothers had no formal education and were not working (i.e., housewives). Moreover, more than half (59.3%) of the families had low household monthly income (<YER20,000; US$1 = YER214). Most of the houses were made of stones and mud whereas few houses are made of burned bricks; only one fifth and a quarter of the houses had piped water supply and electricity, respectively. During the visits to the villages, we observed that toddlers and young children were playing and swimming in the water streams and pools. Many other people were observed using this water for different purposes such as washing clothes and utensils, washing cars and motorcycles and watering animals.

### Prevalence and distribution of schistosomiasis

Urine and faecal samples were collected from 400 children and examined for the presence of *Schistosoma* species and other parasites. A total of 339 (84.8%) children were found to be infected with at least one parasite species. Besides *S. mansoni*, *Ascaris lumbricoides*, *Trichuris trichiura*, *Ancylostoma duodenale*, *Hymenolepis nana*, *Enterobius vermicularis*, *Taenia* sp., *Fasciola* sp., *Entamoeba histolytica/dispar*, *Giardia duodenalis* and *Blastocystis* sp. were also detected in the faecal samples examined. The prevalence and intensity of schistosomiasis according to *Schistosoma* species are shown in Table 2. Of the 400 participants, 127 (31.8%) were found to be infected by either *S. mansoni* or *S. haematobium*. Out of these infected children, 3.9% had mixed infections (i.e., both *Schistosoma* species). Overall, the prevalence of *S. haematobium* infection was higher than *S. mansoni* (23.8% vs 9.3%). The prevalence of schistosomiasis was significantly higher among children aged >10 years compared to those aged ≤10 years (37.6% vs 27.0%; χ^2^ = 5.135; *P* = 0.023). Similarly, male children had higher prevalence of schistosomiasis than females (33.6% vs 29.0%). However, the difference was not statistically significant (χ^2^ = 0.942; *P* = 0.332). With regards to the intensity of infections, 22.1% and 8.1% of *S. haematobium* and *S. mansoni* infections respectively were of heavy intensities (Table 2).

**Table 2:** Prevalence and intensity of schistosomiasis among Yemeni children who participated in this study (n = 400).

Intensity of infection*	Type of infection					
	*S. haematobium*			*S. mansoni*		
	N	%	Mean (ep10ml)	N	%	Mean (epg)
Light	74	77.9	17	19	51.4	50
Moderate	-	-	-	15	40.5	212
Heavy	21	22.1	340	3	8.1	637
Overall	95	23.8	89	37	9.3	163

* According to WHO [18]. ep10ml, Number of eggs per 10 ml of urine. epg, Number of eggs per gram of faeces.

### Clinical manifestations of schistosomiasis

Children who participated in this study underwent physical examination and haemoglobin level was measured. Hepatosplenomegaly and anaemia were reported in 9.5% (38/400) and 39.5% (158/400) of the children, respectively. Moreover, 15.8% (63/400) had fever whilst 24.1% (96/400) had diarrhea. Of these studied children, 26.0% (104/400) and/or 15.0% (60/400) claimed to have haematuria and bloody stool, respectively. The association between schistosomiasis and the presence of hepatosplenomegaly and anaemia was examined. Children with *S. mansoni* infection had a significantly higher rate of hepatosplenomegaly (18.9%; 95% CI = 9.5, 34.2) when compared with those without *S. mansoni* infection (8.3%; 95% CI = 5.8, 11.4) whereas no significant difference in the case of *S. haematobium* infection. A significant association between the intensity of *S. mansoni* infection and hepatosplenomegaly was also reported (*P* = 0.033). Moreover, the presence of hepatosplenomegaly was significantly higher among children with mixed infection (both *Schistosoma* species) compared to those with single infection (*P*>0.05). On the other hand, the association between schistosomiasis and anaemia among these children was not significant (*P*>0.05).

### Factors associated with schistosomiasis

Results of univariate and multivariate analyses for the association of schistosomiasis with demographic, socioeconomic, environmental and behavioural factors are shown in Tables 3 and 4.

**Table 3:** Univariate analysis of factors associated with schistosomiasis among Yemeni children who participated in this study (n = 400).

Variables	Schistosomiasis			
	No. examined	Infected n (%)	OR(95% CI)	*P*
**Age**				
>10 years	178	37.6	1.6 (1.1, 2.5)	0.023*
≤10 years	222	27.0	1	
**Gender**				
Male	238	33.6	1.2 (0.8, 1.9)	0.332
Female	162	29.0	1	
**Fathers' educational levels**				
Non educated	191	38.2	1.8 (1.2, 2.7)	0.008*
Educated (at least primary education)	209	25.8	1	
**Fathers' occupational status**				
Farmers	195	33.8	1.3 (0.8, 2.0)	0.236
Not working	34	38.2	1.6 (0.7, 3.4)	0.233
Government employees & professionals	171	28.1	1	
**Mothers' occupational status**				
Farmer and/or daily labourer	21	47.6	2.0 (0.8, 4.9)	0.109
Not working	279	30.9	1	
**Household monthly income**				
<20,000 YER (low)	253	38.7	2.6 (1.6, 4.2)	<0.001*
≥20,000 YER	147	19.7	1	
**Family size**				
≥8 members (large)	210	28.6	0.7 (0.5, 1.1)	0.151
<8 members	190	35.3	1	
**Presence of toilet in house**				
No	180	39.4	1.9 (1.2, 2.9)	0.003*
Yes	220	25.5	1	
**Source of drinking water**				
Unsafe source (stream, rain, well,..etc)	279	36.2	2.1 (1.3, 3.4)	0.004*
Safe source (pipe)	121	21.5	1	
**Source of household water**				
Unsafe source (stream, rain, well,..etc)	287	35.2	1.8 (1.1, 3.0)	0.018*
Safe source (pipe)	113	23.0	1	
**Presence of stream/spring**				
Yes	125	41.6	1.9 (1.2, 3.0)	0.004*
No	275	27.3	1	
**Presence of pool/pond**				
Yes	80	51.3	2.9 (1.7, 4.7)	<0.001*
No	320	26.9	1	
**Presence of dam**				
Yes	82	35.4	1.2 (0.7, 2.1)	0.430
No	318	30.8	1	
**Presence of water pumps**				
Yes	134	23.1	0.5 (0.3, 0.8)	0.009*
No	266	36.1	1	
**Indiscriminate defecation/urination**				
Yes	245	33.9	1.3 (0.8, 2.0)	0.251
No	155	28.4	1	
**Play/bath in open water source**				
Yes	235	34.0	1.3 (0.8, 2.0	0.240*
No	165	28.5	1	
**Washing clothes or utensil in open water source**				
Yes	119	31.1	1.5 (0.7, 3.3)	0.332*
No	43	23.3	1	
**Foreigners seen play/swim in open water sources**				
Yes	142	23.2	0.5 (0.3, 0.8)	0.007*
No	258	36.4	1	
**Presence of infected family member**				
Yes	140	50.7	3.7 (2.4, 5.8)	<0.001*
No	260	21.5	1	
**Wearing shoes when go outside**				
No	73	37.0	1.3 (0.8, 2.3)	0.288
Yes	327	30.6	1	
**History of schistosomiasis**				
Yes	205	34.6	1.3 (0.9, 2.0)	0.204
No	195	28.7	1	

YER, Yemen Rial; (US$1 = YER 214). OR, Odds ratio. CI, Confidence interval.

* Significant association (*P*<0.05).

**Table 4:** Multivariate analysis of factors associated with schistosomiasis among Yemeni children participated in this study (n = 400).

Variables	Schistosomiasis		
	Adjusted OR	95% CI	*P*
Age	1.4	0.88, 2.33	0.152
Fathers' educational level	1.5	0.91, 2.56	0.106
Household monthly income	2.3	1.33, 3.83	0.003*
Presence of infected family member	4.1	2.40, 6.85	<0.001*
Presence of toilet	1.4	0.77, 2.38	0.288
Source of drinking water	2.5	1.36, 4.41	0.003*
Source of household water	1.1	0.51, 2.44	0.792
Presence of stream/spring	2.1	1.24, 3.63	0.006*
Presence of pool/pond	2.5	1.39, 4.43	0.002*
Presence of water pumps	0.6	0.36, 1.09	0.099
Foreigners seen play/swim in open water sources	0.6	0.47, 1.10	0.052

OR, Odds ratio. CI, Confidence interval.

* Significant key risk factors (*P*<0.05).

Table 3 shows that children aged >10 years (37.6%; 95% CI = 30.8, 44.5) had significantly higher prevalence of schistosomiasis when compared with those aged ≤10 years (27.0%; 95% CI = 21.6, 33.2). Similarly, the prevalence of schistosomiasis was significantly higher among children of non educated fathers (38.2%; 95% CI = 31.6, 45.3) and those from families with low household monthly income (38.7%; 95% CI = 32.9, 44.9) when compared with the children of fathers with at least 6 years of formal education (25.8%; 95% CI = 20.4, 32.2) and those from families with household monthly income of ≥YER20,000 (19.7%; 95% CI = 14.1, 26.9). Moreover, it was found that the presence of other family members infected with schistosomiasis showed significant association with higher prevalence of schistosomiasis (*P*<0.001).

Moreover, children who lived in houses without toilets (39.4%; 95% CI = 32.6, 46.7), those who use unsafe sources for drinking water (36.2%; 95% CI = 75.0, 85.1), those who lived in houses where water used for household purposes was fetched from unsafe sources (e.g., stream, rain, well, water collection tank, trough, etc) (36.2%; 95% CI = 30.8, 42.0) had higher prevalence of schistosomiasis when compared to those having toilets in their houses (25.5%; 95% CI = 20.1, 31.6), those who use piped water (21.5%; 95% CI = 57.2, 69.1) and those living in houses with safe sources of household water (21.5%; 95% CI = 15.1, 29.7).

Furthermore, the results showed that the prevalence of infection was significantly higher among children who lived nearby stream and/or spring (41.6%; 95% CI = 33.3, 50.4) and nearby pool and/or pond (51.3%; 95% CI = 40.5, 61.9) when compared to their counterparts. Interestingly, there was strong negative associations between the presence of water pump and visits by foreigners to the area as the children who lived in the presence of nearby water pump (23.1%; 95% CI = 16.8, 31.0) and those from villages where foreigners were seen playing/swimming in the open water sources (23.2%; 95% CI = 17.0, 30.9) had significantly lower prevalence of infection compared to those who lived in houses not close to water pump (36.1%; 95% CI = 30.6, 42.0) and those from villages where no foreigners were seen playing/swimming in the open water sources (36.4%; 95% CI = 30.8, 42.5).

Five factors associated significantly with schistosomiasis were retained by multiple logistic regression model analysis (Table 4). The presence of other family member infected with schistosomiasis increased the children's odds for the disease by 4.1 times (95% CI = 2.40, 6.85). Similarly, children who used unsafe sources for drinking water had significantly higher odds of having schistosomiasis when compared to those living in houses supplied with piped water (OR = 2.5; 95% CI = 1.36, 4.41). Moreover, children from families with low household monthly income (<YER20,000) had significantly higher odds of schistosomiasis when compared with those from families with higher household monthly income (OR = 2.3; 95% CI = 1.33, 3.83). Furthermore, significantly higher odds of having schistosomiasis were identified among children who lived nearby stream/spring (OR = 2.2; 95% CI = 1.24, 3.63) and those who lived nearby pool/pond (OR = 2.5; 95% CI = 1.39, 4.43) when compared to their counterparts.

## Discussion

Schistosomiasis remains a life-threatening public health problem in many developing countries particularly in rural communities [1]. The present study reported that the prevalence rate of schistosomiasis among children in rural Yemen was 31.8%. This prevalence is consistent with other previous studies carried out in Yemen [14], [17], [24]. A higher prevalence of schistosomiasis (58.9%) was reported among children from Khamir district, Amran province [16]. The present study was carried out in areas undergoing active control and, therefore, the prevalence of 31.8% is considered alarmingly high. A significant reduction in the prevalence rates of schistosomiasis have been reported after 4 campaigns were implemented during 2002–2007 using school-based drug distribution and focal mollusciciding [16]. Subsequently, the prevalence rate increased again and prevalence rates of 30%–60% have been reported from different areas in 2003–2010 [17]. Nowadays, both urinary and intestinal schistosomiasis are endemic in all provinces of Yemen with an estimated overall prevalence of 14%–49% [3], [4].

For the best of our knowledge, the surveillances and control measures were intermittent or ceased in certain areas, other than the area of the present study, from mid 2011through mid 2012 due to the uprising situation in the country. Hence, the prevalence of schistosomiasis is expected to increase. The country is suffering from severe water depletion and this makes people in regular contact with open water sources such as streams, uncovered pools, tanks, cement cisterns/troughs to fetch water for drinking and for domestic use. Moreover, dozens of dams, for agricultural irrigation and also for groundwater recharge, have been constructed by the government throughout the country.

An interesting finding of the present study was the overlap distribution of *Schistosoma* species in Yemen as both main species (i.e., *S. mansoni* and *S. haematobium*) were detected among the studied children. We found that 3.9% of the infected children had mixed infections with Ibb province having the highest prevalence of *S. mansoni* (30.1%) whereas the prevalence of *S. haematobium* was highest in Taiz and Sana'a followed by Hodiedah (36.0%, 36.0% and 33.3%, respectively). Overall, our findings showed that *S. haematobium* was by far more common than *S. mansoni* (accounting for 75% of the *Schistosoma* infections reported). By contrast, a recent study among children from Taiz province showed that *S. mansoni* was more prevalent than S. *haematobium* (20.7% vs 7.4%) [17]. Similar findings were reported among schoolchildren in Ibb province [14].

The geographic distribution of each *Schistosoma* species is closely dependent on the presence of appropriate freshwater snails that serve as the obligatory molluscan hosts. Both genus *Bulinus* and *Biomphalaria* are found in Yemen with *Bulinus* having more species and wider distribution than *Biomphalaria* [25]–[27]. In the present study, the water sources were observed to harbour snails of different genera and with varying degree of snail infestation. Among the provinces, Ibb was the most infested with *Biomphalaria* species followed by Taiz whereas *Bulinus* species was observed more in Sana'a and Hodeidah followed by Taiz. We also observed that water streams of Taiz, Ibb and Hodiedah were more infested with snails compared to water pools, dams and troughs in Sana'a and Dhamar. This may be due to the fact that the streams containing adequate vegetation favour the intermediate host to flourish as compared with the pools and dams.

The findings of the current study also showed that almost a quarter and one fifth of *S. haematobium* and *S. mansoni* infections, respectively, were of heavy intensities. Moreover, 40.5% of the *S. mansoni* infections were of moderate intensity. This percentage of moderate-to-heavy infections is alarmingly high especially considering the fact that clinical manifestations and other complications of this infection are associated with the intensity of infection [28], [29]. In the present study, a high prevalence of anaemia (39.5%) was reported among these children. However, the association between schistosomiasis and anaemia was not statistically significant. Significant associations of heavy schistosomiasis with anaemia, malnutrition and a dismal learning capacity and poor work performance have been reported [30]–[33]. On the other hand, we found that 9.5% of the children had hepatosplenomegaly and its occurrence was significantly associated with *S. mansoni* infection. Similar observations have been reported earlier among children [34]–[36]. However, the association between mixed schistosomiasis (both *Schistosoma* species) and hepatosplenomegaly was not significant. Other potential aetiological agents of hepatosplenomegaly such as visceral leishmaniasis, chronic hepatitis viruses B and C are also prevalent among young children in rural Yemen [4], [37], [38].

The present study showed that children aged >10 years were more prone to be infected than younger children. This is in agreement with previous reports from Yemen and other countries [6], [14], [39]–[41]. This could be explained by the excessive mobility of children at this age and they may become more exposed to infected water while swimming/playing or fetching water for domestic purposes or helping in agriculture activities. With regards to gender, the present study found no significant difference in the prevalence of schistosomiasis between male and female participants. However, we found that boys had significantly higher intensity of both *Schistosoma* species than girls. These are consistent with many other reports in other countries [42], [43]. Males usually have higher prevalence rates of schistosomiasis than females and this was attributed to religious and cultural reasons or to water contact behavior [14], [15], [39], [41], [44]. However, significantly higher infection rates among females compared to their males counterparts have been also reported elsewhere [45], [46]. In Yemen and many other Islamic countries, females are prohibited from bathing in open water sources whereas the males frequently play and swim during their leisure time. On the other hand, females are responsible of fetching water and washing clothes and utensils at these water sources, and therefore, have similar exposure to infective stages. Female education remains a key challenge and gender gap in education in Yemen is among the highest in the world [47]. Hence, community-based drug distribution should also be considered together with the school-based control in order to reach this group and reduce the transmission in the entire communities.

The present study is the first to provide information about the key factors associated with schistosomiasis in Yemen. We found significant associations between the high prevalence of schistosomiasis and the age of children, presence of other family member infected with schistosomiasis, fathers' educational level, household monthly income, lacking toilets and piped water supplies in the households, living nearby streams, pools, water pumps, and living in areas where foreigners seen play/swim in open water.

The findings of the present study showed that children who live in houses with the presence of other family members infected with *Schistosoma* species were at a 4 folds higher risk of getting schistosomiasis. Thus, screening and treating other family members should be considered in the control measures. To the best of our knowledge, no previous study has reported on the association of the presence of other family members infected with *Schistosoma* as a risk factor for schistosomiasis. Although the disease is not transmitted directly from human-to-human but members of a same family may share their activities at water sources such as playing, swimming and washing and therefore, they have similar exposure to the source of infection. Moreover, an infected family member may contract the disease and then contribute to its transmission at the open water sources nearby where other family members may also use.

The association between schistosomiasis and water contact is well documented. The fetching of water and living close to a stream and/or a water pool were identified as significant risk factors for schistosomiasis in the present study. Similar findings have been reported in previous studies among rural children and adolescents in different countries [40], [41], [48], [49]. Water storage, streams, dams and pools may all provide favourable breeding sites for snails and therefore, potentially, support the continued transmission of schistosomiasis in these areas.

Schistosomiasis is a poverty-related disease and our findings showed that children belong to families with a low household monthly income were 2.3 times more likely to be infected compared to those belonging to families with a household monthly income of ≥YER20,000. We have also identified fathers' educational level as a significant predictor of schistosomiasis among the children studied; however, this association was not retained by the logistic regression model. Previous studies among rural communities in Yemen found no association between the prevalence of schistosomiasis and the fathers' or participants' educational status [14], [16]. In Cote d'Ivoire and Nigeria, the higher education level of the head of family was identified as a protective factor against *S. haematobium* infection [39], [40].

In the present study, the absence of a functioning toilet in the house was significantly associated with the prevalence of schistosomiasis and this was in accordance with previous studies [18], [50], [51]. A similar significant association of schistosomiasis with using unsafe water for drinking and for other household purposes was reported in the present study. This association is related to the higher exposure to the infected water during the fetching process.

Surprisingly, there were strong negative associations between schistosomiasis and the presence of a water pump nearby, and living in areas where foreigners were seen playing/swimming in open water sources. The water pump is usually used to provide drinking water or water for agriculture and therefore, people living close to and fetching water for their needs from a water pump are at lower exposure to the infected water in streams and/or pools. Areas where foreigners might be seen frequently are tourist areas and therefore, expected to undergo a better level of cleanliness and services including mollusciciding. However, these significant associations were not retained by the multivariate analysis.

Population migration such as rural-urban migration, forced displacement and the rise of ecotourism may extend the disease to new areas or may cause a shift in snail population especially when the migration is accompanied with some water development projects. Moreover, most of the foreign visitors to these areas, mostly in Ibb province, were from Saudi Arabia and many Yemeni immigrants to USA or UK. Although Saudi Arabia has achieved the elimination of schistosomiasis in 2002, new cases are still reported in southern region, border areas with Yemen [52]. Therefore, cross-border collaboration and regional control programmes are essential, with regular long-term surveillance to detect and treat any new or residual infections [52], [53]. A previous study among a group of 129 Israelis of Yemeni origin found that *S. mansoni* eggs and specific anti- *S. mansoni* IGE were reported positive in 12% and 37% individuals, respectively [54]. In earlier report among 218 Yemeni workers in the San Joaquin Valley of California, eggs of *S. mansoni* were detected in 56% of them with 16% and 27% had heavy and moderate infections, respectively [55]. The authors showed that those who returned to Yemen for short visits had significantly higher egg count compared to those who were away from Yemen for more than 5 years.

Rural communities in Yemen share similar socioeconomic and health profiles with a different climate. Coastal plains and foothills (Taiz, Ibb and Hodeidah) have more streams whereas mountainous areas (Sana'a and Dhamar) have more water pools/troughs and dams. Our study provides a community-based knowledge of schistosomiasis status among children with a poor socioeconomic, environmental and personal hygiene background. Thus, we may speculate that the findings of the present study can be generalised to rural areas in other provinces in Yemen. However, further investigations are required to confirm these conjectures.

### Conclusion

This study reveals an alarmingly high prevalence of schistosomiasis among rural children in Yemen and this supports an urgent need to re-evaluate the current control measures and implement an integrated, targeted and effective schistosomiasis control measures. Regional control programmes are essential to prevent the dissemination of the infection to new areas at neighbouring countries. Screening of other family members and treating the infected individuals should be adopted by the public health authorities in combating this infection in these communities. Besides periodic drug distribution, health education regarding good personal hygiene and good sanitary practices, provision of clean and safe drinking water, introduction of proper sanitation are imperative among these communities in order to curtail the transmission and morbidity caused by schistosomiasis.

## Supporting Information

Checklist S1 STROBE Checklist.
(DOC)
